# Technique-based inoculation against real-world misinformation

**DOI:** 10.1098/rsos.211719

**Published:** 2022-05-18

**Authors:** Jon Roozenbeek, Cecilie S. Traberg, Sander van der Linden

**Affiliations:** Department of Psychology, School of the Biological Sciences, University of Cambridge, Downing Street, CB2 3EB Cambridge, UK

**Keywords:** misinformation, inoculation theory, gamification, fake news, cross-protection

## Abstract

In recent years, numerous psychological interventions have been developed to reduce susceptibility to misinformation. Inoculation theory has become an increasingly common framework for reducing susceptibility to both individual examples of misinformation (issue-based inoculation) and to the techniques and strategies that are commonly used to mislead or misinform people (technique-based inoculation). In this study, we address two open questions related to technique-based inoculation in two separate experiments (total *n* = 2188; convenience sample recruited via the *Bad News* online game platform): (i) can technique-based inoculation effectively reduce susceptibility to real-world misinformation that went viral on social media? and (ii) can technique-based inoculation confer cross-protection against misinformation that does not make use of any of the techniques against which people were inoculated? We find that playing a 15 min game confers psychological resistance against real-world misinformation that makes use of manipulation techniques against which people were inoculated (Cohen's *d* = 0.37, Cohen's *U*_3_ = 64.4%, *p* < 0.001), and that cross-protection is achieved but at a reduced effect size (*d* = 0.10, *U*_3_ = 54.0%, *p* = 0.001).

## 1. Introduction

Online misinformation is a pernicious problem that has proven to be difficult to eradicate with the help of detection algorithms, legislation, and fact-checking initiatives [1,2]. During the COVID-19 pandemic, misinformation and conspiracy theories have proliferated widely online and have been linked to diminished willingness to follow health guidance measures and reduced intentions to get vaccinated [3,4]. In response, researchers have sought to investigate how insights from psychological and behavioural science may be used to address the issue [5,6]. Simple measures such as infographics [7] and pausing for a few seconds to consider the accuracy of a particular news item [8] have shown promise. Recent work on psychological ‘inoculation’ against misinformation has also been fruitful in demonstrating a reduction in individual susceptibility to misinformation [9–11].

Inoculation theory [12] posits that pre-emptively exposing individuals to a weakened form of a misleading argument as well as teaching individuals how to refute those arguments triggers the production of ‘mental antibodies’. This process has been shown to confer psychological resistance against future manipulation attempts, much like a medical vaccine induces resistance against a particular pathogen [13,14]. Inoculation messages typically consist of motivational threat and refutational pre-emption: the threat component forewarns individuals that they may be exposed to a persuasive attack, and refutational pre-emption either entails directly providing individuals with the counterarguments that refute incoming (mis)information, known as *passive* inoculation, or it actively involves the participant in the generation of those counterarguments, known as *active* inoculation [15,16]. Active inoculation may confer a comparatively stronger inoculation effect because people are encouraged to generate their own antibodies and counterarguments [17,18]. That is, as the participant takes an active role in the inoculation process, this allows them to generate internal refutations themselves, which may lead to longer lasting effects owing to increased cognitive involvement [19].

Although early inoculation research focused primarily on ‘cultural truisms’ where participants all had the desired attitude on a given topic, later research has evidenced that inoculation can bolster resistance to persuasion even when people are already familiar with the topic or have been exposed to the misinformation before [20,21], so-called ‘therapeutic inoculation’. Therapeutic inoculation mirrors recent advances in medicine where therapeutic vaccines can still boost the production of antibodies even when people have already been infected [22,23]. A seminal meta-analysis by Banas & Rains [24] highlights the ability of inoculation interventions to confer resistance against persuasive attacks with an average intervention effect size of *d* = 0.43. Several recent reviews have also highlighted the efficacy of inoculation in the context of misinformation specifically [15,16,25].

A final and important distinction has been made between refutational-same versus refutational-different messages [18], where refutational-*same* messages inoculate against specific material individuals will later be exposed to, whereas refutational-*different* messages tackle arguments that individuals may not directly be directly exposed to later, but that may help bolster resistance towards various different persuasive attacks [16]. In recent years, researchers have started to focus on inoculating people against misinformation *techniques,* rather than individual examples of misinformation, representing a form of refutational-different inoculation [16].

The benefit of technique-based and active inoculations lie in both their potential scalability and applicability [16,26,27], as they prepare individuals to resist being persuaded by messages that may be different in *content*, but use the same underlying persuasion *strategy.* While there is some evidence that traditional inoculation messages offer cross-protection (that is, protect the individual against persuasion attempts on untreated, but related attitudes; see [28]), technique-based inoculation potentiates a much a wider level of protection: the fast-paced nature of online misinformation means that the misleading *content* is topically varied and constantly evolving, and therefore it is arguably more effective to target the persuasive techniques which largely remain the same.

**Figure 1.**
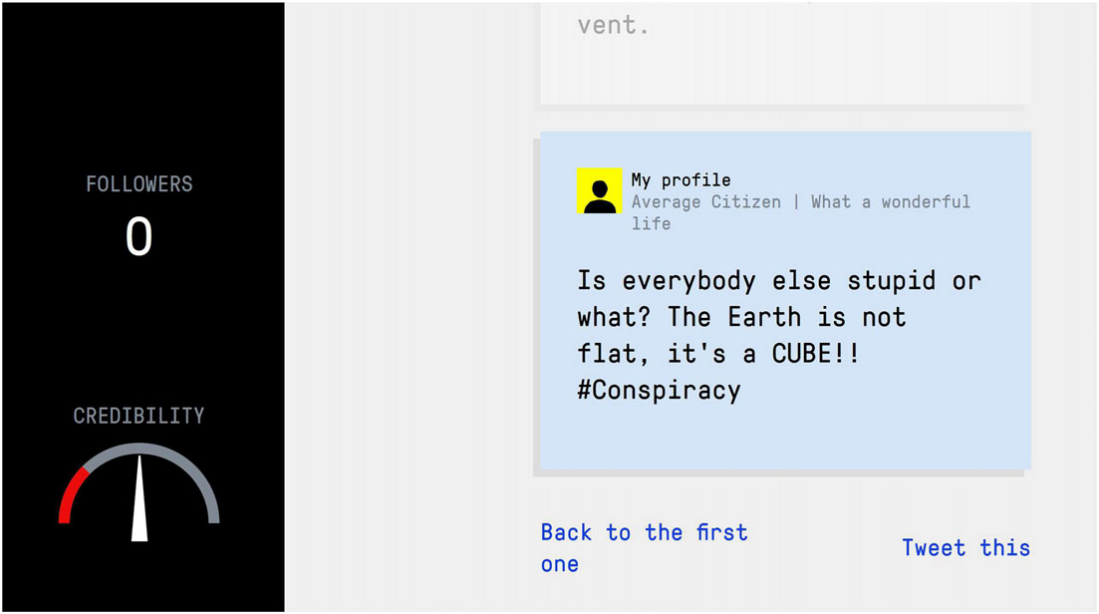
*Bad News* screenshot, with the ‘Followers’ and ‘Credibility’ meters on the left.

One example of an active, technique-based inoculation intervention is *Bad News*,^1^ a free online browser game in which players learn about six common misinformation techniques, a categorization known as **DEPICT**: **D**iscrediting opponents, **E**motional language use, increasing intergroup **P**olarization, **I**mpersonating people through fake accounts, spreading **C**onspiracy theories and evoking outrage through **T**rolling [1,9]. In the game, players take on the role of a fake news creator and are tasked with building a fake news empire by (i) gaining as many followers as they can and (ii) maximizing their credibility. During the game, players are forewarned about the threat of misinformation and exposed to weakened doses of the strategies used in its production, consistent with the mechanisms of inoculation theory [9,29]. Figure 1 shows a screenshot of the game environment.

In a series of studies, *Bad News* was shown to improve people's ability to spot manipulative social media posts [27,30], confer similar effects across different language versions of the game [31], improve people's confidence in their ability to spot misinformation [32] and induce detectable inoculation effects for up to 13 weeks after gameplay if players are given regular reminders or ‘booster shots’ [9].

The efficacy of *Bad News* and other technique-based inoculation interventions has been primarily evaluated by asking people to rate the reliability of fictitious social media posts, designed by the researchers themselves [33]. Participants were shown a series of posts that were designed to make use of one of the manipulation techniques featured in the game (or not, to examine the effect of the inoculation on people's perceptions of ‘real news’), before and after gameplay. Importantly, the misinformation posts were not selected for their truth value per se: while some may contain a kernel of truth, the primary selection criterion has been whether a post uses a particular misinformation technique.

However, several questions about the effectiveness of technique-based inoculation remain unanswered, which we address in this study. First, there is limited data available about whether active inoculation interventions also confer psychological resistance against real-life (as opposed to fictitious) examples of misinformation [17,26]. Second, no studies have directly compared the efficacy of technique-based inoculation for reducing susceptibility to misinformation that is simply *false* (i.e. a headline makes a claim of fact that is probably incorrect but not otherwise manipulative) versus misinformation that makes use of a specific misinformation *technique* that people are inoculated against (but may not contain a claim of fact, such as conspiratorial reasoning). Third, the most common outcome measures for testing technique-based inoculation interventions have been the perceived reliability [9,32] or manipulativeness [17,34] of both misinformation and real news items. As Pennycook *et al*. [35] point out, it is also important to assess whether inoculation improves people's ability to assess the *accuracy* of real and false news. Asking participants to assess the *reliability* or *manipulativeness* of an item does not necessarily mean asking them to assess its veracity (i.e. if it is true or false), whereas this is the case for eliciting a headline's *accuracy*. Importantly, Roozenbeek *et al.* showed that eliciting different questions and response modes should not yield major variations in response patterns when assessing misinformation susceptibility [36].

Resolving these matters is important for several reasons; while using realistic yet fictitious items as outcome measures was crucial in early research to preclude the possibility of memory confounds (i.e. it is impossible for study participants to have seen the item set before, which could otherwise bias their assessment), it also reduces the ecological validity of the evaluation method [9,27]. Testing whether an intervention improves people's ability to spot *real-world* misinformation should therefore be a key indicator of its effectiveness. Furthermore, in a meta-analysis, Walter & Murphy [2] found that correcting real-world misinformation is more challenging than constructed misinformation. Therefore, investigating whether active inoculation interventions can also be effective at preventing unwanted persuasion attempts found in real-world misinformation gives insight into how inoculation interventions can be used to pre-emptively counter such misinformation.

In addition, although researchers have suggested that the benefit of these interventions is their potential to offer broader ‘blanket’ or ‘umbrella’ protection [13,15,28] against a wider array of misinformation, this has yet to be formally tested. As this test is crucial for knowing the extent to which inoculation treatments can confer psychological resistance even against entirely unknown examples of misinformation, the current study aims to examine whether and to what extent technique-based inoculation interventions can offer cross-protection against misinformation that inoculated individuals were not trained to recognize.

Finally, different question framings (e.g. *reliability* versus *accuracy*) should not be expected to measure substantially different constructs, and so we may expect broadly similar response patterns. Assessing whether the effects of technique-based inoculation interventions are robust to different methods of measurement is an important indicator of the overall efficacy of such interventions.

## 2. The present research

In the light of the above questions, this study is to our knowledge, the first to evaluate *Bad News* using stimuli found ‘in the wild’ in two separate experiments. Following previous research designs by Roozenbeek & van der Linden [27] and Roozenbeek *et al*. [30], we implemented two voluntary pre-post survey experiments within the *Bad News* game environment. In experiment 1, we use an item set with social media posts that make use of one of the **DEPICT** techniques, to test if the *Bad News* game successfully reduces susceptibility to real-world misinformation that uses a misinformation technique they learned about in the game. In experiment 2, we use a different item set of false and true headlines used in previous studies [37,38] that do not explicitly make use of a **DEPICT** technique, to test whether cross-protection against untreated misinformation is achieved [28]. In addition, in experiment 2, we ask about the *accuracy* rather than the *reliability* of a set of headlines.

Between 15 March and 7 September 2020, a total of 2 188 valid completed survey entries were collected (*n_1_* *=* 1,216, *n_2_* = 972). The rationale for the data collection period was twofold. First, based on previously known effect sizes in similar studies (Cohen's *d* = 0.30), a power analysis determined a minimum of 483 participants would be necessary to capture previously known effect sizes of approximately *d* = 0.30, and second, the availability of the online platform hosting the study. This study was approved by the Cambridge Psychology Research Ethics Committee (PRE.2019.103). The datasets, measures, items and our visualization scripts are available on the OSF: https://osf.io/59pjk/. We discuss both experiments separately below.

## 3. Experiment 1

### 3.1. Method

**Figure 2.**
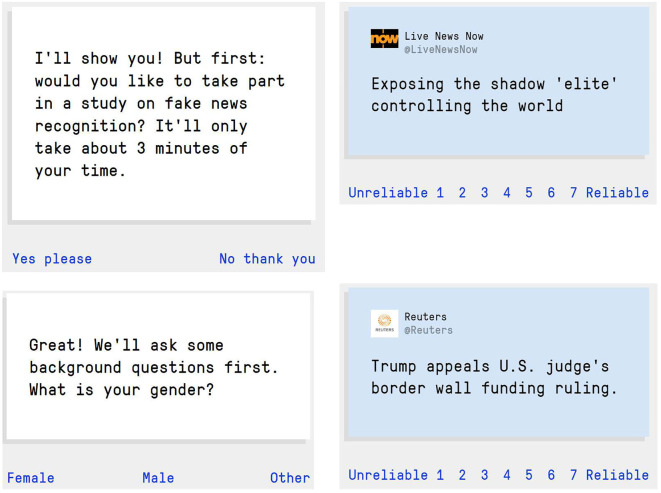
Examples of the *Bad News* in-game survey environment for experiment 1. Clockwise from top left: start of the survey; a misinformation post (using the ‘conspiracy’ technique); a real news (control) post and a demographic question.

Following Roozenbeek & van der Linden [27], experiment 1 is a simple within-subjects pre-post survey, administered at the start and at the end of the *Bad News* game. Survey participants were asked to rate the reliability of a series of nine social media posts on a 1–7 Likert scale, with 1 being ‘unreliable’ and 7 being ‘reliable’, at the start and end of the game. They were also asked several demographic questions (age, gender, education level and political affiliation). Figure 2 shows screenshots of the in-game survey environment.

Two real news (control) posts were taken from credible news sources, Reuters and Associated Press [39,40], and did not contain any misinformation, such as ‘Trump appeals U.S. judge's border wall funding ruling’ (figure 2). The other six (misinformation) items were real examples of misinformation and matched so that each made use of one of the manipulation techniques people are inoculated against in the game: discrediting, emotion, polarization, impersonation, conspiracy, and trolling [1]. For example, the item making use of emotionally evocative language read ‘Horrific TV show inspiring suicide, says filmmaker’, and the item making use of the conspiracy technique read ‘Exposing the shadow ‘elite’ controlling the world’ (figure 2). In addition, we also included one false item about the novel coronavirus [4,41,42].^2^ See the electronic supplementary material, table S2 for the full item wordings.

The misinformation items were a combination of previously used headlines by Traberg & van der Linden [43] and new headlines taken from an online fact-checking platform developed by researchers at Indiana University known as Hoaxy [44]. All items were selected based on their use of the **DEPICT** misinformation techniques mentioned above and were converted into a Tweet-format to facilitate integration in the game environment (figure 2). For the impersonation and trolling techniques, no ecologically valid examples were found through Hoaxy. The reason for this is that impersonating people or organizations online [45] and trolling (i.e. baiting people on social media into responding emotionally or lashing out at others, see [46]), while common, are not featured on Hoaxy as examples of misinformation. We therefore relied on a manual Twitter search as well as previous (credible) news reporting of online trolling scams to find examples of social media content that made use of these techniques. In total, participants were thus shown nine social media posts before and after playing *Bad News* (two control items, one item for each of the six techniques, and one item about COVID-19). Participants were shown the same items pre- and post-gameplay. The items and their sources can be found in the electronic supplementary material, table S2. In experiment 1, we test the following hypotheses:

**H_1_:** people who play *Bad News* rate real-life examples of misinformation [**H_1a_**], but not real news [**H_1b_**] as significantly less reliable after playing; and
**H_2_:** people who play *Bad News* improve in their ability to discern the reliability of real-life examples of manipulative and non-manipulative news.

### 3.2. Sample

A total of 1 216 valid responses were collected. The sample consists of 55.1% men, with 48.0% of survey participants reporting being between 18 and 29 years old,^3^ with 58.0% having obtained a higher degree. Participants were also somewhat left-leaning (*M* = 3.29, s.d. = 1.43 on a 7-point Likert scale, 1 being ‘very left-wing’ and 7 being ‘very right-wing’). See the electronic supplementary material, table S1 for the sample composition.

### 3.3. Results

Following Roozenbeek & van der Linden [27], we conduct a series of paired-samples *t*-tests on the pre- and post-scores for each social media post (item), as well as on the aggregated scores for the misinformation and real news (control) items (see the electronic supplementary material, tables S3 and S4). For the misinformation items (averaged across all seven items), we find a significant reduction in perceived reliability post-gameplay (*M*_pre_ = 2.58 versus *M*_post_ = 2.22, *M*_diff_ *=* −0.35, *t*_1215_ = −12.79, *p* < 0.001, *d* *=* −0.37, 95% confidence interval (CI) [−0.43, −0.31]). This translates to 64.4% of the post-gameplay reliability scores being lower than the mean of pre-gameplay reliability scores (i.e. Cohen's *U*_3_ = 64.4). We also find that participants rate all six misinformation posts that made use of one of the manipulation techniques learned in *Bad News* as significantly less reliable after playing (all *p*'s < 0.001, with Cohen's *d* ranging between *d* = −0.12 and *d* = −0.27). In addition, participants rated the post about coronavirus as significantly less reliable after gameplay (*M*_pre_ = 1.88 versus *M*_post_ = 1.78, *M*_diff_ *=* −0.10, *t*_1215_ = −2.05, *p* = 0.04, *d* *=* −0.06, 95% CI [−0.12, −0.003]), albeit with a substantially lower effect size. We note that the pre-score for the coronavirus post is the lowest out of all the items and is even lower than the lowest post-gameplay score for the other items (*M*_coronavirus,pre_ = 1.88 versus *M*_discredit,post_ = 1.90), indicating possible flooring effects (see the electronic supplementary material, table S4).

In order to test whether the inoculation effect is different across different levels of initial (i.e. pre-gameplay) levels of misinformation susceptibility, we also grouped people (in terciles) by their performance on the pre-test. Comparing across reliability judgements on the pre-test, we find that the effect of inoculation was most pronounced for those who were most susceptible to misinformation prior to gameplay (*F*_2,2426_ = 64.8, *p* < 0.001, *η*^2^ = 0.025), a finding consistent with results from Roozenbeek & van der Linden [27]. The full overview of this analysis can be found in the electronic supplementary material, table S5; see also figures S2 and S3. Overall, our results thus support hypothesis **H_1a_**.

Second, we find that participants also rate ‘real’ news as significantly less reliable after playing, albeit with a lower effect size than misinformation (*M*_pre_ = 5.35 versus *M*_post_ = 5.18, *M*_diff_ *=* −0.17, *t*_1215_ = −3.55, *p* < 0.001, *d* *=* −0.10, 95% CI [−0.16, −0.045]). However, we note that we only used two real news posts, and that only one post shows a significant pre-post difference (*p*_real,Brexit_ < 0.001 versus *p*_real,Trump_ = 0.15), potentially indicating that item effects are at play. Nonetheless, we do not find support for hypothesis **H_1b_**_._

Third, we find a significant difference *between* the difference scores (real news minus misinformation reliability scores) before and after gameplay (*M*_diff,pre_ = 2.78 versus *M*_diff,post_ = 2.96, *M*_diff,diff_ *=* 0.18, *t*_1215_ = 3.45, *p* < 0.001, *d* *=* 0.10, 95% CI [0.042, 0.15]), indicating improved truth discernment. These results support hypothesis **H_2_**.

To see to what extent demographic variables influence the inoculation effect, we conduct a linear regression with the average pre-post different scores for the misinformation (false news) items as the dependent variable and gender, age, education and political affiliation as independent variables. We find that none of these variables significantly predict the pre-post-inoculation effect for the false news items (all *p*'s > 0.387), except for education level (*p* = 0.007), so that people with a higher education level also display a larger inoculation effect.

Finally, we also conducted a linear regression with the same independent variables, but with the difference in discernment (i.e. mean reliability scores for the real news (control) posts minus mean reliability scores for the misinformation posts) before and after playing *Bad News* as the dependent variable. We find that none of the independent variables are significant predictors of improved discernment (all *p*'s > 0.206); see the electronic supplementary material, table S6.

**Figure 3.**
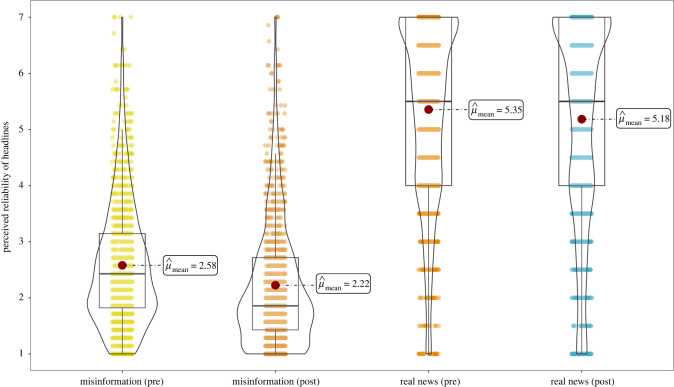
Pre-post RDI plot for misinformation items pre- and post-gameplay, and the real news (control) headlines pre- and post-gameplay. Pre-post differences are significant for both misinformation (*t*_1215_ = −12.79, *p* < 0.001, *d* *=* −0.37) and real news (*t*_1215_ = −3.55, *p* < 0.001, *d* *=* −0.10). See the electronic supplementary material, table S3 and figure S1 for item-level results.

Figure 3 shows the results from experiment 1 in a raw data, description and inference (RDI) plot.

### 3.4. Discussion

Overall, we find that playing *Bad News* significantly decreases the perceived reliability of real-world misinformation that made use of one of the **DEPICT** misinformation techniques. The effect sizes are in line with the initial within-subject study on the effectiveness of *Bad News* [27], which used a similar methodology to the present study; Roozenbeek & van der Linden [27] report pre-post within-subjects effect sizes between *d* = −0.16 and *d* = −0.36 for their fictitious social media posts, whereas we find slightly smaller effects in this study: between *d* = −0.06 for the coronavirus item, which suffers from flooring effects in the sense that almost all participants found this post highly unreliable already in the pre-test (*M*_pre_ = 1.88 versus *M*_post_ = 1.78), and *d* = −0.27 for the ‘emotion’ item. Nonetheless, the average effect size for all misinformation posts of *d* = −0.37 is encouraging.

Second, in line with recent findings on the effectiveness of media literacy interventions [7], we find that playing *Bad News* slightly decreases the perceived reliability of real news alongside misinformation, albeit to a much smaller extent (*d* = −0.37 versus *d* = −0.10). However, we note that we used a smaller number of real news than misinformation items and are therefore unable to rule out that the observed findings are owing to item effects [30]. The fact that only one out of two real news items showed a significant reduction in perceived reliability post-gameplay supports this notion. In addition, truth discernment [37,38] increased significantly after gameplay, indicating that *Bad News* players improve in their ability to discern real information from misinformation.

## 4. Experiment 2

### 4.1. Method

In experiment 2, we used the two-group within-subject design developed by Roozenbeek *et al*. [30] in order to address several open questions not answered in experiment 1. First, while the items in experiment 1 consisted of social media posts, in experiment 2, participants were shown a series of real and false news headlines (including headline source) as they may appear on someone's social media feed. These headlines were selected from a larger popular set of true and false headlines used in previous research on misinformation (see [37,38]). The headlines were selected based on their representativeness of different forms of misinformation that individuals may be exposed to online, and not necessarily their internal consistency.^4^

Second, in experiment 1, participants were shown the same items before and after gameplay. Following Roozenbeek *et al*. [30], participants in experiment 2 were therefore shown different sets of real and false headlines before and after playing (we will call these sets A and B). At the start of the survey, participants were randomly assigned to a group (A-B or B-A). The A-B group was shown item set A before gameplay, and item set B after gameplay. The B-A group was first shown set B and then set A. Doing so allows us to (i) compare the accuracy ratings of real and false headlines for the *same* item sets (A-A and B-B) by *different* participants before and after gameplay; (ii) compare the accuracy ratings for *different* item sets (A-B and B-A) for the *same* participants and (iii) examine the overall effect (averaged across both item sets) for the real and false headlines, as well as for truth discernment.

**Figure 4.**
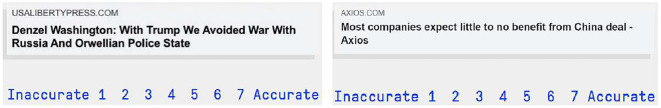
Examples of the *Bad News* in-game survey environment for experiment 2, with a false headline (left) and a true headline (right) adapted from Pennycook & Rand [37,38].

Third, following Pennycook *et al*. [35,37,47], we asked participants to rate the *accuracy* (rather than the reliability) of real and false news headlines on a 1–7 Likert scale (1 being ‘inaccurate’ and 7 being ‘accurate’). Fourth, as pointed out by Maertens *et al*. [9], in order to more accurately assess whether playing *Bad News* improves truth discernment (i.e. people's ability to discern true from false news), it is useful to present an equal number of real and false items. Participants were therefore shown four real and four false headlines before gameplay, and a different set of four real and four false headlines after gameplay, for a total of eight real and eight false headlines. Figure 4 shows screenshots of what these headlines looked like in the game environment (see the electronic supplementary material, table S7 for the full item sets).

Again following Roozenbeek *et al*. [30], we test the following hypotheses:

**H_1_:** when comparing an index of the same items, pre-test (group x) with post-test (group y), there is a significant decrease in the perceived accuracy of false headlines, both for item set A-A [**H_1a_**] and item set B–B [**H_1b_**];
**H_2_:** when comparing an index of the same items, pre-test (group x) with post-test (group y), there is no significant decrease in the perceived accuracy of real headlines, both for item set A-A [**H_2a_**] and item set B–B [**H_2b_**];
**H_3_:** when comparing different item sets, pre-test (group x) with post-test (group x), there is a significant decrease in the perceived accuracy of false headlines, both for group A-B [**H_3a_**] and group B-A [**H_3b_**];
**H_4_:** when comparing different item sets, pre-test (group x) with post-test (group x), there is no significant difference in the perceived accuracy of real headlines, both for group A-B [**H_4a_**] and group B-A [**H_4b_**]; and
**H_5_:** people who play *Bad News* significantly improve in their ability to discern the accuracy of real-life examples of real and false headlines.

### 4.2. Sample

A total of 968 valid pre-post survey responses were collected from the in-game survey. The sample consisted of 50.7% men, with 55.5% of respondents being between 18 and 29 years old, and 51.8% having obtained a higher degree. As in experiment 1, participants were left-leaning (*M* = 3.17, s.d. = 1.39). In addition, we also asked participants about their region of origin (46.4% being from the USA, 26.9% from Europe), and whether participants had played *Bad News* before (10.2% yes, 89.8% no). See the electronic supplementary material, table S1 for the full sample composition.

### 4.3. Results

We first look at whether an inoculation effect is found when crossing the item sets between groups (hypotheses **H_1_** and **H_2_**), comparing item set A (B) pre-test scores with item set A (B) post-test scores. Doing so yields a significant effect for the false news items for item set A (pre-test for the A-B group) versus item set A (post-test for the B-A group) (*M*_Fake,SetA,pre_ = 2.66 versus *M*_Fake,SetA,post_ = 2.41, *M*_diff_ = 0.25, *t*_966_ = 3.109, *p* = 0.002, *d* = 0.20, 95% CI [0.073, 0.33]). These results support hypothesis **H_1a_**. However, we find no significant difference for the false news items for item set B (pre-test for the B-A group) versus item set B (post-test for the A-B group) (*M*_Fake,SetB,pre_ = 2.36 versus *M*_Fake,SetB,post_ = 2.40, *M*_diff_ = 0.04, *t*_966_ = 0.518, *p* = 0.605, *d* = 0.03, 95% CI [−0.093, 0.16]); see the electronic supplementary material, tables S8 and S9. To check whether this result implies the absence of an effect of interest, we performed a two one-sided *t*-test (TOST) equivalence test with a smallest effect size of interest of *d* = (−)0.20 and *α* = 0.01 [48].^5^ Doing so confirms statistical equivalence to zero: *t*_964_ = −2.60, *p* = 0.005. Our data thus does not support hypothesis **H_1b_**.

We perform the same analyses for the real news items. Doing so yields no significant difference for both item set A (*M*_Real,SetA,pre_ = 4.77 versus *M*_Real,SetA,post_ = 4.68, *M*_diff_ = 0.09, *t*_966_ = 1.176, *p* = 0.002, *d* = 0.076, 95% CI [−0.051, 0.20]) and item set B (*M*_Real,SetB,pre_ = 4.36 versus *M*_Real,SetB,post_ = 4.50, *M*_diff_ = 0.14, *t*_966_ = 1.766, *p* = 0.078, *d* = 0.11, 95% CI [−0.013, 0.24]); see the electronic supplementary material, tables S8 and S9. However, A TOST equivalence test does not confirm statistical equivalence to zero for item set A (*t*_924_ = −1.93, *p* = 0.027) or item set B (*t*_961_ = −1.34, *p* = 0.09), which means that we cannot rule out that there is an effect (i.e. an *increase* in the perceived accuracy of real news post-gameplay) that is larger than *d* = 0.20. These results support hypothesis **H_2a_**, and partially support hypothesis **H_2b_**.

Next, we examine whether inoculation effects can be detected when comparing different item sets within the same groups (hypotheses **H_3_** and **H_4_**). When looking at groups A-B and B-A together, we find a significant overall reduction in perceived accuracy post-gameplay for the false news items (*M*_Fake,pre_ = 2.52 versus *M*_Fake,post_ = 2.41, *M*_diff_ *=* 0.11, *t*_966_ = −3.223, *p* = 0.001, *d* = −0.10, 95% CI [−0.17, −0.04]). This means that 54.0% of the post-gameplay false headline accuracy scores are lower than the mean of pre-gameplay accuracy scores (i.e. Cohen's *U*_3_ = 54.0). However, this effect is only significant for the A-B group (*M*_Fake A-B,pre_ = 2.67 versus *M*_Fake,A-B,post_ = 2.41, *M*_diff_ *=* 0.26, *t*_512_ = −5.544, *p* < 0.001, *d* = −0.24, 95% CI [−0.33, −0.16]), but not for the B-A group (*M*_Fake,B-A,pre_ = 2.36 versus *M*_Fake,B-A,post_ = 2.41, *M*_diff_ *=* 0.05, *t*_454_ = 0.956, *p* = 0.34, *d* = 0.05, 95% CI [−0.047, 0.14]); see the electronic supplementary material, tables S8 and S9. A TOST equivalence test confirms statistical equivalence to zero for the B-A group: *t*_454_ = 3.31, *p* < 0.001. We thus find support for hypothesis **H_3a_**, but not for hypothesis **H_3b_**.

For the real news items, we find no significant overall difference in perceived reliability post-gameplay (*M*_Real,pre_ = 4.58 versus *M*_Real,post_ = 4.58, *M*_diff_ *=* 0.007, *t*_966_ = 0.173, *p* = 0.86, *d* = 0.01, 95% CI [−0.057, 0.069]). However, for the A-B group, we find a significant *decrease* in perceived reliability of real news (*M*_Real A-B,pre_ = 4.77 versus *M*_Real,A-B,post_ = 4.50, *M*_diff_ *=* 0.27, *t*_512_ = −4.789, *p* < 0.001, *d* = −0.21, 95% CI [−0.23, −0.12]), whereas for the B-A group, we find the opposite, namely a significant and descriptively larger *increase* in the perceived reliability of real news (*M*_Real B-A,pre_ = 4.36 versus *M*_Real,B-A,post_ = 4.68, *M*_diff_ *=* 0.32, *t*_454_ = 5.279, *p* < 0.001, *d* = 0.25, 95% CI [0.15, 0.34]); see the electronic supplementary material, tables S8 and S9. We thus find no support for either hypothesis **H_4a_** or hypothesis **H_4b_**.

**Figure 5.**
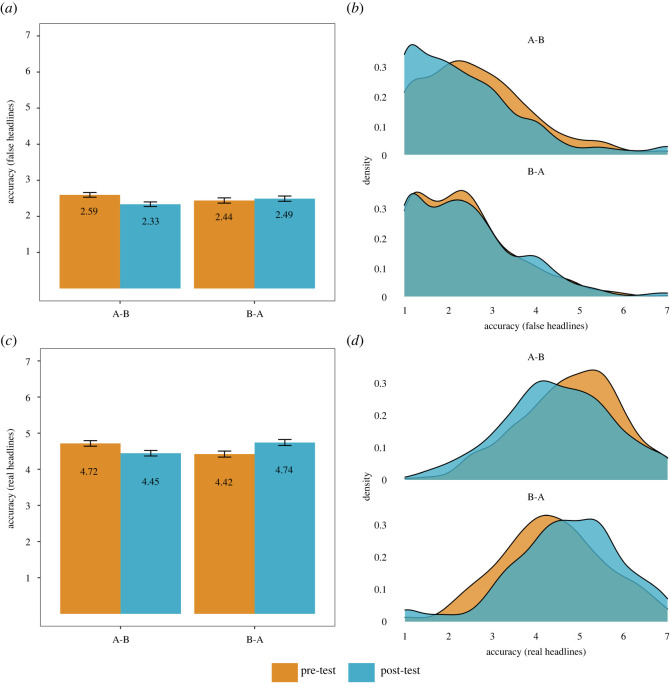
Bar graphs and density plots for false headlines (*a,b*) and real headlines (*c,d*). *Note: n* = 968. Error bars represent the 95% confidence interval.

In terms of participants' ability to distinguish real and false news headlines, we find a significant overall increase in truth discernment post-gameplay, indicating that *Bad News* players generally improve in their ability to discern true from false information (*M*_Discernment,pre_ = 2.06 versus *M*_Discernment,post_ = 2.18, *M*_diff_ *=* 0.12, *t*_966_ = 2.421, *p* = 0.016, *d* = 0.08, 95% CI [0.015, 0.14]). These results support hypothesis **H_5_**; see the electronic supplementary material, tables S8 and S9. Figure 5 shows the results in a bar graph and density plot for both the false and real headlines.

Finally, as in experiment 1, we check whether any covariates influence the observed inoculation effects. To do so, we again first conduct a linear regression with the average pre-post different scores for the misinformation (false news) items as the dependent variable, group (A-B or B-A) as a factor variable, and gender, age, education and political affiliation as independent variables. Doing so shows that none of our covariates significantly predicts the pre-post-inoculation effect for false headlines (all *p*'s > 0.063). Second, we conduct a linear regression with the same factor and independent variables, but with the difference in truth discernment before and after gameplay as the dependent variable. Doing so shows that none of the covariates predict the pre-post-inoculation effect for truth discernment (all *p*'s > 0.14), except age (*p* = 0.005), so that being older is associated with a higher pre-post difference in truth discernment; see the electronic supplementary material, table S10.

### 4.4. Discussion

Overall, we find that playing the *Bad News* game significantly decreases the perceived accuracy of false headlines, with no effect on real headlines, leading to improved truth discernment. However, this effect was only significant for the A-B group, and not for the B-A group. Furthermore, while the overall effect for real news headlines was not significant, we found a significant pre-post *reduction* in the perceived accuracy of real news for the A-B group and a significant *increase* in perceived accuracy for the B-A group. These findings indicate that the experiment might suffer from item effects, in the sense that the observed results may be skewed by the items that were used: rather than the *Bad News* game influencing the perceived accuracy of real headlines, we find that the observed pre-post differences are owing to imbalances in the *initial* perceived accuracy of headline sets A and B [30]. This is to be expected, as the item sets were not psychometrically validated *a priori*. Nonetheless, our findings are highly encouraging for the general applicability of *Bad News* as a tool to learn how to spot misinformation, especially because the false headlines used in this experiment did not explicitly make use of the misinformation techniques learned in the game. Overall, our results show a reduced (but still significant) inoculation effect even for misinformation that *Bad News* players were not inoculated against, indicating partial cross-protection [28].

## 5. General discussion and conclusion

Across two large-scale experiments, we find that the *Bad News* game confers psychological resistance against misinformation techniques used in real-life examples of online misinformation. In line with previous research, we find that players' truth discernment, or their ability to distinguish manipulative from non-manipulative information, significantly improves after gameplay. Importantly, this effect is primarily driven by a decreased perceived reliability (or accuracy) of misinformation. For real news, the findings are somewhat more ambiguous; while in experiment 1, we find a small but significant decrease in perceived reliability, we find the same effect for one item set used in experiment 2, but the opposite in the second item set, namely an *increase* in the perceived accuracy of real news (in line with findings by, for example, Pennycook *et al*. [49], who reported a post-intervention increase in participants’ willingness to share real news headlines). In general, these results align with the findings by Roozenbeek *et al*. [30].

Although not a consistent finding (and one that may be owing to item effects), the observed decrease in the perceived reliability of real news warrants further discussion, as this is not an explicit goal of the inoculation intervention. A similar phenomenon was observed for a different anti-misinformation intervention, so-called ‘digital literacy tips' [7]. We argue that this finding is not a major concern, for two reasons. First, so long as (i) a more accurate identification of misinformation is the main psychological target (that is, that we see a significant difference between the reduced reliability of misinformation versus real news, which is the case here) and (ii) the overall reduction in perceived reliability of real news remains small, this finding is arguably not alarming. For example, while participants may have reduced their reliability judgements of real news in experiments 1 and 2, their overall judgement was that real headlines were still on the very high end of the reliability scale, meaning they did not change their judgement entirely from reliable to unreliable. Second, believing any news headline from a single source to be 100% reliable may not be the ideal way to make accurate judgements about news headlines in general, as sometimes even mainstream media sites publish misleading information, perhaps owing to human error [50]. That is, when it comes to news media consumption in the real world, it may in fact be beneficial to rely on multiple sources before accepting a news headline to be entirely true, as all sources may have their own spin on current events [51].

With respect to false news, while our findings in experiment 1 are straightforward, the results from experiment 2 are more ambiguous: effect sizes reported in this experiment are descriptively smaller than in experiment 1, and group B-A showed no significant decrease in the perceived accuracy of false headlines [30]. That said, the overall results still point towards an improved ability to distinguish real from false headlines. These results are in line with findings by Walter & Murphy [2], who reported that real-world misinformation is more difficult to correct than constructed misinformation.

One reason for this non-predicted effect may be that the false headlines we used in experiment 2 did not make use of the **DEPICT** misinformation techniques learned while playing *Bad News* (e.g. using emotional language, trolling or impersonation), and players were not specifically inoculated against these types of false headlines (as the intervention does not seek to improve people's ability to distinguish between false and true information, but rather aims to improve people's ability to recognize the use of specific manipulation techniques). This may explain the differences between the present study and those reported in Roozenbeek *et al*. [30], who did include items that made use of misinformation techniques featured in the game. Nonetheless, the fact that we still find a significant improvement in truth discernment and reduced overall accuracy for false headlines is encouraging, as it shows that the inoculation effect conferred by playing the *Bad News* game is at least partially transferrable to previously unseen types of misinformation. In other words, active inoculation treatments appear to be at least somewhat effective at conferring cross-protection against related but untreated persuasive attacks [13,15,28]. Though we note that there is ongoing discussion on the boundaries between the ‘umbrella’ or ‘blanket’ of protection (which confers resistance against different examples of the *same* misinformation technique) versus true *cross*-protection, which could occur when resistance is formed against novel misinformation that either does not make use of the same strategies (as in the current study) or makes more explicit use of entirely different strategies (akin to different topics) that people were not inoculated against. Future research will have to examine—in a more controlled way—whether inoculation games can also confer *cross-protection* in the sense that inoculating people against one misinformation technique (e.g. conspiracy theories) also confers protection against unmentioned and entirely different techniques (e.g. impersonation).

Although we conducted two studies, we were still unable to account for several key limitations. First, with respect to covariates, although we do not find political ideology to be an independent predictor of the inoculation effect (see the electronic supplementary material, table S9), previous research has found political similarity with sources to impact misinformation susceptibility [43]. As such, it is still possible that more complex political factors involving the political slant of sources or news content may have impacted the inoculation effect found in experiment 2 (as these headlines included source information).

Second, our item sets were not psychometrically validated *a priori*, and we note the presence of item effects that may complicate our findings. We were only able to use a limited number of items in each experiment, and the low internal consistency for our item sets, especially in experiment 2, limits the generalizability of our findings. Third, this study also explored whether eliciting different question framings (e.g. the reliability or accuracy of an item set) can be expected to yield different response patterns when testing the efficacy of anti-misinformation interventions. Our results are inconclusive: although we find that both question framings yield significant results in the hypothesized direction, the effect sizes in experiments 1 and 2 are substantially different. However, because we used different item sets, we are unable to directly compare both experiments' response patterns. Thus, while this study is to our knowledge, the first to test two separate outcome measures (reliability and accuracy) on the same intervention, it is unclear to what extent these measures are interpreted differently by participants, and how this may affect their item ratings; see Roozenbeek *et al.* [36].

Finally, more can be said about the mechanisms underlying the efficacy of active inoculation games. In related research, scholars have provided some early evidence for the role of motivational threat [52], which proved significantly higher in active than passive inoculation treatments [17]. Other fruitful avenues include the potential role of confidence and attitude certainty [32] as well as higher post-inoculation sharing following active versus passive inoculation [17]. We suggest that future research further explores the potential cognitive and affective mechanisms by which active inoculation interventions can confer resistance to misinformation, particularly insofar it (i) makes people aware of their own vulnerability and (ii) elicits greater motivation to protect oneself from manipulation [53].

Overall, we corroborate previous findings that playing *Bad News* improves people's ability to spot misinformation [27,30,32]. This is especially important in light of the game's relatively easy scalability (being free-to-play in a browser on a phone, computer or tablet) and availability in approximately 20 languages. The game may be implemented as part of media literacy curricula in schools, or played as a standalone game in-browser. In addition, the game may be deployed in conjunction with other anti-misinformation tools such as videos [54], ‘prebunking’ infographics [17], accuracy-based interventions [8,47] or media literacy interventions [7], to improve resilience against online misinformation at scale.

## Data Availability

The datasets, measures, items and our visualization scripts are available on the OSF: https://osf.io/59pjk/. Electronic supplementary material is available online at [55].

## Footnotes

1 Playable at www.getbadnews.com.

2 Although the items were not selected based on their conceptual correlation (given that they rely on different misinformation techniques), we do note that the seven misinformation items show internal consistency (*M =* 2.59, s.d. *=* 1.16, Cronbach's *α =* 0.74). The real news (control) items have relatively low internal consistency (*M =* 5.35, s.d. *=* 1.58, Cronbach's *α =* 0.52), most likely owing to the low number of items, so we report item-level as well as averaged results.

3 Participants under 18 were excluded, as per our ethics approval.

4 We find low internal consistency (*M*_SetA,Real_ *=* 4.77, s.d._SetA,Real_ *=*1.15, Cronbach's *α =* 0.38; *M*_SetA,Fake_ = 2.67, s.d._SetA,Fake_ *=* 1.26, Cronbach's *α =* 0.62; *M*_SetB,Real_ *=* 4.36, s.d._SetB,Real_ *=* 1.19, Cronbach's *α =* 0.59; *M*_SetB,Fake_ *=* 2.36, s.d._SetB,Fake_ *=* 1.11, Cronbach's *α =* 0.55).

5 We used the TOSTER package in Jamovi for equivalence testing. The SESOI of *d* = +−0.20 was based on the effect size of *d* = 0.20 obtained when comparing item set A (pre) to item set A (post). See also: https://daniellakens.blogspot.com/2017/03/equivalence-testing-in-jamovi.html.
