# Correction: ‘Technique-based inoculation against real-world misinformation’ (2023), by Roozenbeek *et al.*

**DOI:** 10.1098/rsos.231235

**Published:** 2023-12-06

**Authors:** Jon Roozenbeek, Cecilie S. Traberg, Sander van der Linden

*R. Soc. Open Sci.* **9**: 211719 (Published online 18 May 2022). (https://doi.org/10.1098/rsos.211719)

We would like to bring to readers' attention an error in our published manuscript; ‘Technique-based inoculation against real-world misinformation’ (https://doi.org/10.1098/rsos.211719). We were alerted to a potential error in the dataset where for one of the items, the pre and post columns were reversed in the original excel file. This did not ultimately matter for the significance or effects reported in the paper but we did have to adjust the means and test statistics. In addition, a duplicate cleaning procedure resulted in two slightly different datasets being produced. Again, this makes no difference to the results at all, but we wanted to provide both datasets on our OSF page for maximum transparency. The datasets are provided with this correction.

## Revised results section (post-correction)

Following Roozenbeek & van der Linden [1], we conduct a series of paired-samples *t*-tests on the pre- and post-scores for each social media post (item), as well as on the aggregated scores for the misinformation and real news (control) items (see electronic supplementary material, tables S3 and S4). For the misinformation items (averaged across all seven items), we find a significant reduction in perceived reliability post-gameplay (*M*_pre_ = 2.59 versus *M*_post_ = 2.21, *M*_diff_ *=* *−*0.38, *t*_1215_ = −11.27, *p* < 0.001, *d* *=* −0.32, 95% CI [−0.27, −0.38]). This translates to 62.6% of the post-gameplay reliability scores being lower than the mean of pre-gameplay reliability scores (i.e. Cohen's *U*_3_ = 62.6). We also find that participants rate all six misinformation posts that made use of one of the manipulation techniques learned in *Bad News* as significantly less reliable after playing (all *p*'s < 0.001, with Cohen's *d* ranging between *d* = −0.12 and *d* = −0.27). In addition, participants rated the post about coronavirus as significantly less reliable after gameplay (*M*_pre_ = 1.88 versus *M*_post_ = 1.78, *M*_diff_ *=* −0.10, *t*_1215_ = −2.05, *p* = 0.04, *d* *=* −0.06, 95% CI [−0.12, −0.003]), albeit with a substantially lower effect size. We note that the pre-score for the coronavirus post is the lowest out of all the items, and is even lower than the lowest post-gameplay score for the other items (*M*_coronavirus,pre_ = 1.88 versus *M*_discredit,post_ = 1.90), indicating possible flooring effects (see electronic supplementary material, table S4).

In order to test whether the inoculation effect is different across different levels of initial (i.e. pre-gameplay) levels of misinformation susceptibility, we also grouped people (in terciles) by their performance on the pre-test. Comparing across reliability judgements on the pre-test, we find that the effect of inoculation was most pronounced for those who were most susceptible to misinformation prior to gameplay (*F*_2,2426_ = 93.1, *p* < 0.001, *η*^2^ = 0.039), a finding consistent with results from Roozenbeek & van der Linden [1]. The full overview of this analysis can be found in the supplement (electronic supplementary material, table S5). See also electronic supplementary material, figures S2 and S3. Overall, our results thus support hypothesis **H_1a_**.

Second, we find that participants also rate ‘real’ news as significantly less reliable after playing, albeit with a lower effect size than misinformation (*M*_pre_ = 5.35 versus *M*_post_ = 5.18, *M*_diff_ *=* *−*0.17, *t*_1215_ = −3.55, *p* < 0.001, *d* *=* −0.10, 95%CI [−0.16, −0.045]). However, we note that we only used two real news posts, and that only one post shows a significant pre-post difference (*p*_real,Brexit_ < 0.001 versus *p*_real,Trump_ = 0.15), potentially indicating that item effects are at play. Nonetheless, we do not find support for hypothesis **H_1b_**_._

Third, we find a significant difference *between* the difference scores (real news minus misinformation reliability scores) before and after gameplay (*M*_diff,pre_ = 2.76 versus *M*_diff,post_ = 2.97, *M*_diff,diff_ *=* 0.21, *t*_1215_ = 3.75, *p* < 0.001, *d* *=* 0.11, 95% CI [0.051, 0.164]), indicating improved truth discernment [2]. These results support hypothesis **H_2_**.

To see to what extent demographic variables influence the inoculation effect, we conduct a linear regression with the average pre–post different scores for the misinformation (false news) items as the dependent variable and gender, age, education and political affiliation as independent variables. We find that none of these variables significantly predict the pre–post inoculation effect for the false news items (all *p*'s > 0.053, for the education variable), see electronic supplementary material, table S6.

Finally, we also conducted a linear regression with the same independent variables, but with the difference in discernment (i.e. mean reliability scores for the real news (control) posts minus mean reliability scores for the misinformation posts) before and after playing *Bad News* as the dependent variable. We find that none of the independent variables are significant predictors of improved discernment (all *p*'s > 0.486); see electronic supplementary material, table S6.

Figure 3 shows the results from experiment 1 in an RDI plot (raw data, description and inference).

Supplementary material is available online [3].

**Figure 3.**
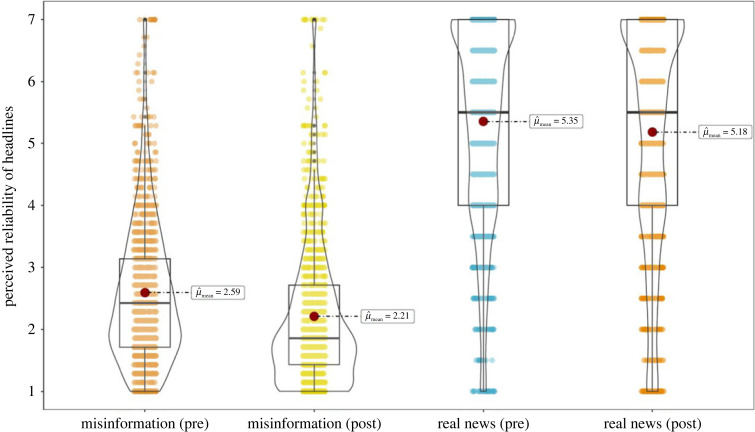
Pre-post RDI plots for misinformation items pre- and post-gameplay, and the real news (control) headlines pre- and post-gameplay. Pre-post differences are significant for both misinformation (*t*_1215_ = −11.27, *p* < 0.001, *d* *=* −0.32) and real news (*t*_1215_ = −3.55, *p* < 0.001, *d* *=* −0.10). See electronic supplementary material, table S3 and figure S1 for item-level results.
